# Switching on lysosomes

**DOI:** 10.7554/eLife.102430

**Published:** 2024-08-21

**Authors:** Deepak Adhikari, John Carroll

**Affiliations:** 1 Development and Stem Cell Program and the Department of Anatomy and Developmental Biology, Monash Biomedicine Discovery Institute, Monash University Melbourne Australia

**Keywords:** fertilization, ELYSA, oocyte, embryo, lysosome, endosome, Mouse

## Abstract

The formation of large endolysosomal structures in unfertilized eggs ensures that lysosomes remain dormant before fertilization, and then shift into clean-up mode after the egg-to-embryo transition.

**Related research article** Satouh Y, Tatebe T, Tanida I, Yamaguchi J, Uchiyama Y, Sato K. 2024. Endosomal-lysosomal organellar assembly (ELYSA) structures coordinate lysosomal degradation systems through mammalian oocyte-to-embryo transition. *eLife* **13**:RP99358. doi: 10.7554/eLife.99358.

Unfertilized eggs, known as oocytes, contain proteins and other macromolecules that are essential for the early development of embryos. These macromolecules are swiftly degraded after fertilization occurs, and then recycled so that their building blocks can be used by the developing embryo, but the process by which they avoid being degraded before fertilization has long puzzled scientists.

Degradation relies on a process called autophagy, which also removes defective or unwanted components from cells (Mizushima and Komatsu, 2011). Autophagy begins with materials in the cytoplasm of the cell being enclosed within membrane-bound vesicles called autophagosomes, which then fuse with endosomes, and subsequently with lysosomes. The materials are ultimately broken down by a combination of enzymes that are activated by the acidic environment within the lysosomes.

Previous work has shown that autophagy is only triggered upon fertilization, and that it is essential for early embryo development in mice (Tsukamoto et al., 2008). The timing of the lysosomal activity in autophagy is also important: if it happens too soon, the oocyte’s reservices will be depleted, but if it happens too late, the embryo will be starved of the basic building blocks it needs to develop. Now, in eLife, Yuhkoh Satouh, Ken Sato and colleagues at Gunma University and Juntendo University report the discovery of giant multi-organelle structures in oocytes, which might hold the key to explaining how oocytes are able to get the timing of lysosomal activity just right (Satouh et al., 2024).

First, the team stained mouse oocytes and used a combination of microscopy techniques (including correlative light and electron microscopy) to reveal the distribution of endosomes and lysosomes within the cells. This showed that these organelles had aggregated to form giant structures in immature oocytes. Moreover, these structures – which were not confined inside a membrane – then migrated to the periphery of the cell in mature oocytes, before falling apart after fertilization (Figure 1). Satouh et al. called these short-lived structures ELYSAs (which is short for endosomal-lysosomal organellar assemblies). Some ELYSAs also expressed the autophagosome marker LC3, which suggests that all of the molecular machinery needed for degradation is present in the structure.

**Figure 1.**
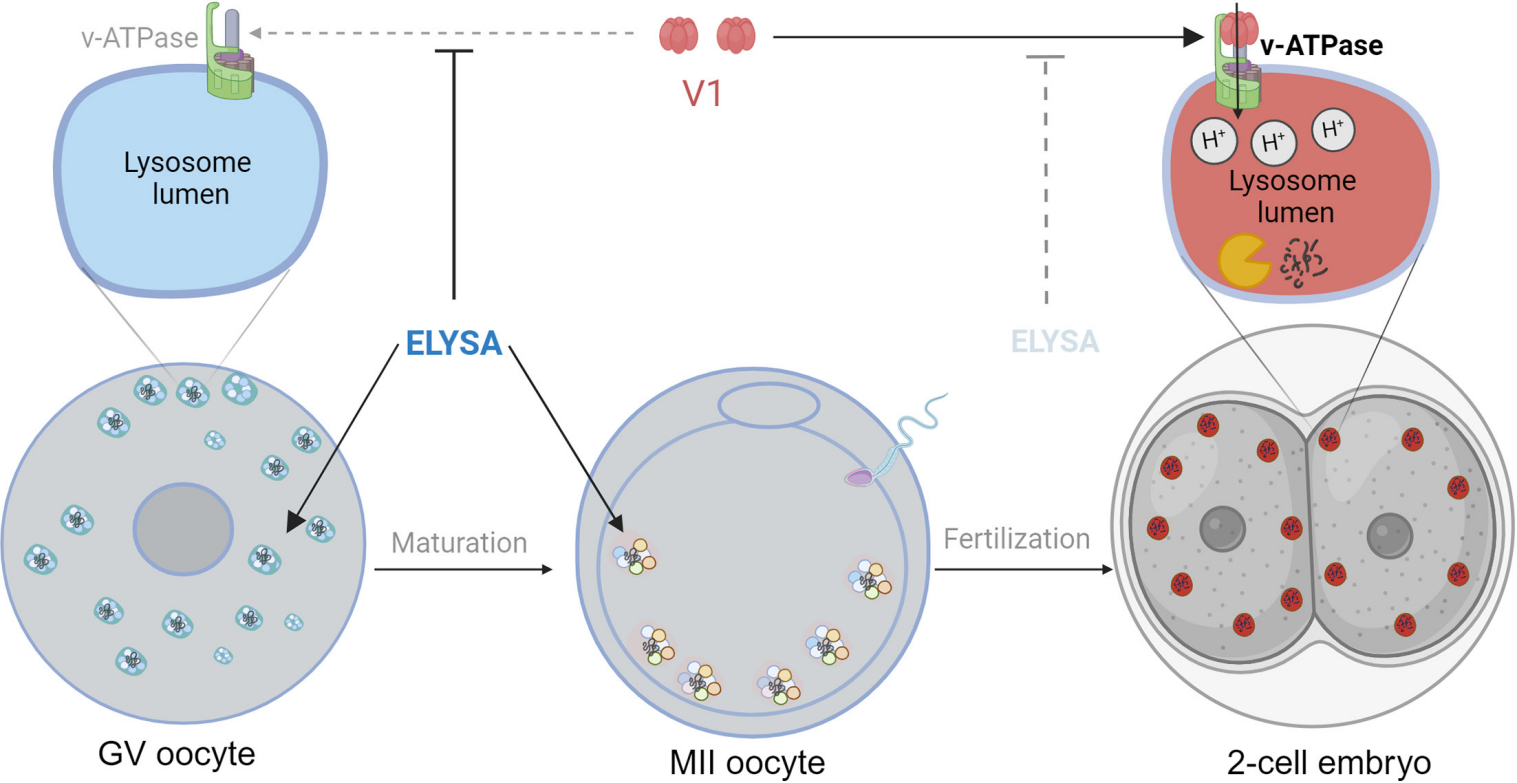
The formation of ELYSAs prevents the acidification of lysosomes in oocytes. In immature oocytes at the germinal vesicle (GV) stage (grey, left), endosomes (white) and lysosomes (blue) assemble in large spherical structures called ELYSAs. The formation of an ELYSA prevents the assembly of v-ATPase (green; top left inset) on the surface of the lysosomes it contains by blocking the recruitment of the V1 subunit (red), and this in turn prevents acidification of the lysosomes. As the GV oocyte matures into an MII oocyte (middle), ELYSAs fuse with one another to form even bigger structures that can measure 7–8 microns across, and these move towards the periphery of the cell. After the MII oocyte has been fertilized, ELYSAs begin to disassemble in the 2 cell stage embryo (right). This allows V1 to bind to v-ATPase (top right inset), which leads to an influx of protons into the lysosomes (red), creating an acidic environment that increases degradation within the lysosomes. ELYSA: endosomal-lysosomal organellar assembly.

However, despite containing lysosomes, the ELYSAs appeared unable to degrade macromolecules, mostly because they were not acidic. The process of acidification is regulated by enzymes known as V-ATPases that actively pump protons into the lumen of lysosomes to maintain an acidic environment. The V1 subunit of the enzyme is particularly important for this process. Satouh et al. found that V1 was not present on lysosomes within the ELYSAs in oocytes, but that the recruitment of V1 to lysosomes began after fertilization (Figure 1). Experiments that measured the acidity of lysosomes also showed that the acidity increased when the ELYSAs started to fall apart in an embryo. This suggests that oocytes assemble structures like ELYSAs to prevent premature acidification of the lysosomes, thus preserving the macromolecules that support the early development of the embryo. Such precise temporal regulation of acidification in oocytes and early embryos is remarkable.

These findings are reinforced by another recent study that independently described similar structures in mouse oocytes (Zaffagnini et al., 2024). These structures – called ELVAs by their discoverers – are involved in sequestering protein aggregates in eggs and facilitating the breakdown of proteins after fertilization.

Interestingly, oocytes appear to employ different strategies for storing proteins with different functions. For example, ELYSAs are used to store proteins that are destined for degradation upon fertilization, whereas structures called cytoplasmic lattices are used to store maternal proteins that persist in the early embryos (Morency et al., 2011; Jentoft et al., 2023; Bomba-Warczak et al., 2024) and are essential for normal embryo development (Li et al., 2008).

The findings of Satouh et al. begin to characterize ELYSAs in late-stage oocytes. However, it remains unknown whether ELYSAs are present from the onset of oogenesis, or if they emerge at a specific point during oocyte growth. It is also unclear if the process of disassembly is initiated by sperm-derived signals, as seen in *Caenorhabditis elegans* (Bohnert and Kenyon, 2017), or by some other process. This could be explored by experimentally activating oocytes through parthenogenesis, thus bypassing fertilization.

In the future, it will be fascinating to explore whether a failure to assemble ELYSAs in oocytes, or an untimely disaggregation of ELYSAs in embryos, affects oocyte and embryo health. The processes discussed above – notably autophagy and maintaining the proton gradient needed for acidification – require energy in the form of ATP. Since mitochondria are crucial for ATP generation in oocytes, and suboptimal mitochondrial fitness is often observed during maternal aging and obesity (Adhikari et al., 2022), studying the impact of these conditions on lysosomal activity during fertilization could be insightful. If so, this research might open new avenues for pharmacological modulation of autophagy pathways, potentially improving embryo quality.
